# Traditional healing practices in rural Bangladesh: a qualitative investigation

**DOI:** 10.1186/s12906-018-2129-5

**Published:** 2018-02-15

**Authors:** Md. Imdadul Haque, A. B. M. Alauddin Chowdhury, Md. Shahjahan, Md. Golam Dostogir Harun

**Affiliations:** 1 Department of Public Health, Daffodil International University, 102 & 102/1 Shukrabad, Mirpur Road, Dhanmondi, Dhaka, 1207 Bangladesh; 2 Health, Nutrition and Population Program, BRAC, Mirpur, Dhaka, 1216 Bangladesh

**Keywords:** Complimentary medicine, Rural community, Traditional healing, Qualitative investigation, Bangladesh

## Abstract

**Background:**

Traditional healing practice is an important and integral part of healthcare systems in almost all countries of the world. Very few studies have addressed the holistic scenario of traditional healing practices in Bangladesh, although these serve around 80% of the ailing people. This study explored distinctive forms of traditional healing practices in rural Bangladesh.

**Methods:**

During July to October 2007, the study team conducted 64 unstructured interviews, and 18 key informant interviews with traditional healers and patients from Bhabanipur and Jobra, two adjacent villages in Chittagong district, Bangladesh. The study also used participatory observations of traditional healing activities in the treatment centers.

**Results:**

Majority of the community members, especially people of low socioeconomic status, first approached the traditional healers with their medical problems. Only after failure of such treatment did they move to qualified physicians for modern treatment. Interestingly, if this failed, they returned to the traditional healers. This study identified both religious and non-religious healing practices. The key religious healing practices reportedly included *Kalami*, *Bhandai*, and Spiritual Healing, whereas the non-religious healing practices included Sorcery, *Kabiraji,* and Home Medicine. Both patients and healers practiced self-medication at home with their indigenous knowledge. *Kabiraji* was widely practiced based on informal use of local medicinal plants in rural areas. Healers in both *Kalami* and *Bhandari* practices resorted to religious rituals, and usually used verses of holy books in healing, which required a firm belief of patients for the treatment to be effective. Sorcerers deliberately used their so-called supernatural power not only to treat a patient but also to cause harm to others upon secret request. The spiritual healing reportedly diagnosed and cured the health problems through communication with sacred spirits. Although the fee for diagnosis was small, spiritual healing required different types of treatment instruments, which made the treatment implicitly expensive.

**Conclusions:**

Traditional healing was widely practiced as the means of primary healthcare in rural areas of Bangladesh, especially among the people with low socioeconomic status. The extent of services showed no decline with the advancement of modern medical sciences; rather it has increased with the passage of time.

## Background

Traditional and complementary medicine (T&CM) is an important and often underestimated issue of healthcare. T&CM is found in almost all countries of the world and the demand for such services is increasing. Traditional medicine (TM) of proven quality, safety, and efficacy, contributes to achieving the goal of ensuring that all people have access to care [1]. According to the World Health Organization (WHO 2013), it is estimated that about 80% of the ailing population in developing countries including Bangladesh depends on traditional healing for their primary healthcare (PHC) needs. What is presently known as ‘conventional medicine’ had its origin in the West [2]. According to the Fifth Plan Document (1992), India has more than half a million traditional healers. This number, of course, includes all practitioners of alternative medicine. A gross estimate suggests that more than 90% of the Indian population uses these traditional healing services at some point in time [3]. The practices of traditional healing are deeply rooted in the cultural heritage of Bangladesh and constitute an integral part of the culture of the people of this country. Different forms of traditional healing practices have been used in this country as an essential means of treatment for diseases and management of various health problems from time immemorial. The practice of traditional healing in this country has flourished tremendously in the recent years, along with that of modern treatment [4]. Traditional healing systems of medical treatment have assumed a unique position in the healthcare of people living in remote areas of the country [5]. Traditional healing practices include not only medicinal substances of natural origin but also items like magic (sorcery), charms, incantations, religious verses, spiritual methods, amulets, sacrifices, rituals, and even invasive physical and mental torture [6]. As this system of treatment has been in use for generations both for various physical and psychological diseases, it is called ‘traditional’ [6].

The traditional healing practices in Bangladesh include mainly folk medicine based on locally-available substances, cultural practices, and religious rites, as well as Ayurvedic and Unani systems based on scientific use of pharmaceutical methods and technology. Traditional and complementary medicine (T&CM) is often used inseparably, and traditional medicine is widely understood as a part of complementary and alternative medicine (CAM) in Bangladesh. However, on an average, only about 20–25% of the people have access to modern healthcare facilities, and the remaining 75–80% of the rural population in Bangladesh still receives healthcare services from the indigenous traditional medical practitioners [4]. The use of these practitioners has greater significance for local people because of the long tradition, reportedly effective outcome, and their long-held belief in its effectiveness [7]. Traditional healing practice is holistic and aims at the overall well-being of the person. It takes the body, self, and society within a framework of dynamic equilibrium. The holistic approach takes into consideration the values, passions, beliefs, social interactions, and spiritual orientation of a person [3]. Although the use of traditional healing practices is the largest in rural areas where access to biomedical health services is lower, it is not the case that traditional healing has lost its importance for healthcare provision in urban areas [8]. In spite of the development of an extensive network of a modern healthcare delivery system all over the country, traditional healing systems still play a significant role in delivering healthcare at the community level [5].

Unani and Ayurvedic systems of medicine were officially recognized by the Government of Bangladesh immediately after independence, and at the same time, the Board of Unani and Ayurvedic Systems of Medicine was constituted [4]. However, in many parts of the world, including Bangladesh, policy-makers, health professionals, and the public at large are struggling with issues regarding the safety, effectiveness, quality, availability, preservation, and regulation of traditional and complementary medicine (T&CM) [1]. All the traditional healers are not officially recognized by the governments in many countries, where they perform healing practices outside the formal health care structures. However, leaving traditional healers on the sidelines can have serious consequences because of the dependence of people on them [9].

The choice of a healer in a medically-pluralistic society is a complex process. It depends on a variety of factors, such as the severity of the disease, patients’ perceived risk of the disease, relative proximity of the healer, cost of healthcare, transportation facilities, gender of the patients, attitude of the patients toward different systems of health care practices, past experience of the patients, perception of illness, and belief system on the causes of disease [10–12]. Like many other developing countries, medical pluralism, or the existence of several distinct therapeutic systems in a single cultural setting, is an important feature of health care in Bangladesh. [13]. Several studies have discovered the use of indigenous medical treatments in modern hospitals in low-income countries, where patients have limited resources [7, 14]. Research on traditional medicine in Bangladesh has also been conducted from different perspectives [7].

Some researchers have conducted studies in rural areas to find out the religious, traditional and cultural practices used in healing different diseases [7]. Most of the earlier researches focused on a single system of traditional healing practices, either a single case study, Unani and Ayurvedic systems, *Kabiraji or* Spiritual healing practice individually. Few studies concentrated on the safety and effectiveness of non-botanical traditional medicine [8, 15]. Very little is known about how multiple traditional healing practices have co-existed in rural Bangladesh over time, using indigenous knowledge, spiritual beliefs and locally available plants and materials**.** Therefore, the key objectives of the study included:

- To explore the distinctive, diversified traditional healing practices based on two most common types: religious and non-religious procedures.
- To investigate realm of rituals, belief systems and indigenous treatment behaviors in resolving specific diseases.

## Methods

### Study design and sampling

The study used 64 unstructured interviews, 18 key informant interviews (KIIs), and 5 participatory observations to understand the depth and breadth of the phenomenon, and for comparing, triangulating, and filling in knowledge gaps about traditional healing systems and types. Of the 18 key informant interviews, 10 were conducted among traditional healers, and 8 among local elites and knowledgeable local people. Qualitative approaches in this study were useful in providing rich descriptions of complex perceptions and belief systems regarding traditional healing practices, tracking unique or unexpected events and giving voice to those whose views are rarely heard [16].

Snowball sampling technique was employed for the recruitment of the study participants. This sampling technique is often used in qualitative research to recruit hidden populations that are difficult for researchers to access [17]. The traditional healers usually do not want to disclose the information relating to their treatment procedures, as traditional healing practices, except the Unani and Ayurvedic systems, are still not officially permitted by the Government.

### Study setting

**Fig. 1:**
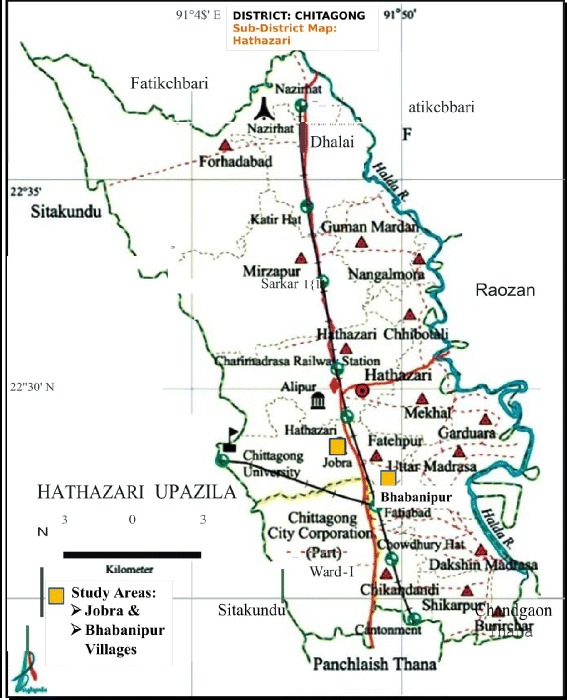
Study Areas: Jobra and Bhabanipur villages in Hathazari sub-district of Chittagong district, Bangladesh [Adapted from Google Map] (Source: https://www.google.com/search?q=map+of+hathazari+upazila&client=firefox-b-ab&source=lnms&tbm=isch&sa=X&ved=0ahUKEwiP2ZbIu6PZAhWBQ5QKHQQaAb4Q_AUIDCgD&biw=1366&bih=611#imgrc=CQ-rx1G8hT93SM:)

The data were collected from Bhabanipur and Jobra villages of Hathazari sub-district under Chittagong district from July to October 2007 (Fig. 1). The area was predominantly rural with nearby peri-urban settlements with high levels of unemployment and poverty and low levels of educational attainment. The population groups of these regions use traditional healers.

### Interview guideline

Keeping in mind the key objectives and research questions of the study, contemporary literature reviews regarding traditional, complementary and alternative medicines, and its healing practices, experiences, rituals and beliefs encompassing traditional healing systems, an unstructured comprehensive interview guide for healers and patients was developed by the research team. Since the interviews in this study took place as informal conversation, the interview guide was used to probe or check the answers given by the traditional healers or patients at the time of interview as well as to support the interviewers by allowing flexibility to vary and deepen interesting aspects. After conducting the first few interviews, the interview guideline was critically reviewed and revised by the research team. [17]. Table 1 shows the interview guideline.

**Table 1:** Interview guideline

Questions to the healers	Questions to the patients
1. Would you please tell me about your socio- economic information (age, religion, level of education, occupation and monthly income)?	1. Would you please tell me about your socio- economic information (age, religion, level of education, occupation and monthly income)?
2. Please tell me, how did you come to be a healer? Recall and inform me in details; what was it like? Can anyone become a healer or do you think it is special gift? What is the source of your healing knowledge?	2. Please describe what you have experienced during the healing process? What did you feel then? Did it make any change in your life? How?
3. For how many years have you been practicing traditional healing?	3. For how many years have you been using the services from traditional healers practicing?
4. Do you consider yourself to be a positive or both positive and negative influence for the patients? If yes/no, how?	4. Have you ever experienced negative effects of traditional healing? If yes, how?
5. Please describe, what type of healing practices do you perform? Please tell me how a healing activity takes place? Maybe you can share an example of recent healing practice? Do you pursue other professions besides healing?	5. Please describe, what type of healing practices do you usually follow for you and for your family? Maybe you can share an example of how have you benefited in healing?
6. Do you prepare yourself for a healing treatment? Where does the healing take places? Who participate in it? Do you follow any specific healing time?	6. Did you prepare yourself for the healing treatment? Where did the treatment take place? Who participates in it?
7. Who usually visits you, how and why? How do people find you?	7. Who do you usually visit, how and why? How do people find a healer? Have you been to other healers before?
8. How would you consider the following: disease, health and cure? What do you think are the causes of disease?	8. How would you consider the following: disease, health and cure? What do you think are the causes of disease?
9. What are the processes you follow for diagnosis a problem?	9. What did you expect from healing treatment? Why did you consult with a healer?
10. What are the usual disorders for which you offer healing? How do you feel about conventional doctors? What are the eligibility criteria you follow to be healed in traditional healing?	10. How are you, since you resorted to the traditional healing system? What exactly has changed? When did the change begin?
11. What are the healing requirements or rituals you suggest for the patients? Do you follow any preventive measures in your healing practices?	11. How do particular beliefs or religions function in healing?
12. How do particular beliefs or religions function in healing?	12. If following a traditional healing does not work as curative, what else you do?
13. Do you apply your healing knowledge to your family or for yourself? Do you wish your children or your next generation to devote themselves to the healing profession?	13. When was the first time you visited a healer? Recall, what was it like? Tell me something about your life and disease history.

### Data collection

The study employed unstructured interviews and in-depth interviews to explore different types of traditional healing practices, community perceptions, reported practices, and social norms relating to traditional healing practices. The study also used participatory observation to explore and understand the actual practice in the real-life settings. All three methods of data collection helped in triangulating the findings. All the interviews and participatory observations took place either in the healing centers of traditional healers or in the home of the patients and sometimes in public spaces in the community.

The first author of this article conducted the interviews and participatory observations; he was academically trained on the methods of data collection and the ways of approaching the participants for gathering data; he was familiar with the local language, as well. All raw data were directly collected in Chittagonian dialect and were tape-recorded. Before commencing data collection, the researchers thoroughly reviewed the research objectives, research tools, and specific data-collection techniques to collect quality data effectively and efficiently. During in-depth interviews, lasting from 45 min to 60 min and unstructured interviews, requiring 55 to 90 min, the researcher used the interview guide that started by asking participants to list all illnesses they could recall that could be healed by the use of traditional practices.

The key informant interviews yielded socioeconomic information on the healers, source/way of getting the healing power, cause of illness, type of the problems presented with, diagnostic procedures to gauge the disorders, methods of healing, instruments or fees required for treatment. However, the interview methods were not always convenient to get the appropriate information on diagnostic procedures for different diseases and their healing methods. In those cases, the researcher approached the healers as a patient and observed their diagnostic procedures for different problems, accompanied with specific healing methods. In particular, participatory observations were helpful to probe into and cross-check with the data from interviews and to know the subjective experiences, biographies, beliefs and motivations of healers and their patients [17].

Informal conversations with 64 patients from traditional healers were taken as unstructured interviews, by which the study gathered information on their socioeconomic background, healing practices, beliefs in cause and prevention of illness, influence of religion in healing, specific health problems for going to the traditional healers or to qualified physicians, effectiveness of traditional healing procedure and modern medical treatment, and how they benefited from the traditional practices. The participants for the KIIs were selected from the most knowledgeable local people, personnel of non-governmental organizations, government officials, local, neutral and social activists, school teachers and traditional healers, with similar socio-demographic backgrounds and representative of the participants from the study community. Participants for unstructured interview were recruited with a reference who used, benefited or was affected by the tradition healing practices in their lifetime. Some potential and knowledgeable participants in rural areas were busy with their regular farming and other activities, and the interviews sometimes required a long time to complete. At least two return visits were made to interview the respondents to ensure that no participants dropped out from the interview once it started.

### Data analysis

Collected textual data from unstructured interviews, KIIs, and participatory observations were transcribed in standard Bangla from tape-recoded Chittagonian dialect; notes that were taken during interviews were also used in the analysis. Intra-cultural standard for translation was followed in understanding the local values, beliefs, and cultural practices regarding illness, along with healthcare-seeking behavior, and, finally, all the data were edited by a medical anthropologist in the research team, who belonged to Chittagong region. The data were classified and rearranged theme-wise with relevant quotations, then coded manually according to the research objectives, and finally checked by the supervisor of the study on a daily basis. Considerable diversity along with merging the similar concepts into common themes and subthemes were followed in subtracting the codes and developing the theme [18]. After coding, those data were translated into English and thematic content analysis was performed in order to provide a descriptive results. Although data from each KII, unstructured interview, and participatory observation were analyzed separately, inferences were drawn collectively in the results. Key findings (Heading) in the study were presented by type of traditional healing practices in the rural areas, and sub-heading included religious and non-religious healing types, who and when go to the traditional healer, services offered by traditional healers, functions of religions in healing, and prevention measures. Socio-demographic characteristics of the traditional healers and patients were presented in simple frequencies. All the authors significantly contributed in distributing contents, orderly classification of data and in writing the final report.

### Ethical consideration

The objectives and methods used in the study were explained to all participants prior to starting data collection. Written informed consent was obtained from the participants before interview to participate in the study, and they were assured that all their personal information would be preserved with confidentiality. In addition, the participants were informed that their participation was voluntary and could be terminated at any time without reason and without any penalty. People appeared to be spontaneous and felt free in giving their information to the interviewer because they were informed about publication of their way of healing practices, which they considered to be valuable. The research evaluation committee of the Department of Anthropology, University of Chittagong, Bangladesh, approved the study.

## Results

### Socioeconomic characteristics of the participants

Mean age of traditional healers was 46 (SD ±5.9) years, with minimum 24 years and maximum of 78 years. Half of them (5/10) had no formal education; slightly more than one-third (4/10) were women. Half (5/10) were Hindus, and monthly income of the majority of traditional healers (8/10) was <BDT10,000; one-fifth (2/10) of them were full-time healers (Table 2). Around two-thirds (50/72) of the patients from traditional healers were female, and their mean age was 33 (SD ±4.5) years; three-fourths (55/72) of them were 18–45 years of age. Half of the patients from traditional healers (36/72) were housewives, In terms of education, nearly three-fourths (51/72) had only up to primary-level education. (Table 3).

**Table 2:** Socio-demographic characteristics of traditional healers**)**

Socio-demographic Characteristics		*n* = 10	%
Sex	Male	6	60
	Female	4	40
Age	18–39 years	1	10
	40–59 years	5	50
	60 years and above	4	40
Religion	Islam	4	40
	Hinduism	5	50
	Buddhism	1	10
Occupation	Full-time healer	2	20
	Agriculturist	2	20
	Housewife	4	40
	Small businessmen	2	20
Monthly income (in BDT)	< 10,000	8	80
	10,000–20,000	1	10
	> 20,000	1	10
Level of education	No formal education	5	50
	Primary	3	30
	Secondary/Higher secondary	2	20
Source of traditional healing knowledge	Mentoring by another healer	5	50
	Forefather	3	30
	Indigenous	2	20
Type of practices	Religious healing (*Kalami/Bhandari*/Spiritual)	7	70
	Non-religious healing (Sorcery/ *Kabiraji*/ Home medicine)	3	30
Year of practices	< 5 years	1	10
	5–10 years	2	20
	> 10 years	7	70

**Table 3:** Socio-demographic characteristics of patients from traditional healers

Socio-demographic Characteristics		*n* = 72	%
Sex	Male	22	31
	Female	50	69
Age	18–30 years	7	9
	31–45 years	48	67
	45 years	17	24
Religion	Islam	27	38
	Hinduism	37	51
	Buddhism	8	11
Occupation	Agricultural worker	11	15
	Day laborer	8	11
	Housewife	36	50
	Small business men	12	17
	Government and non-government service-holder	5	7
Monthly income (in BDT)	< 10,000	45	63
	10,000–20,000	19	26
	> 20,000	8	11
Level of education	No formal education	27	38
	Primary	24	33
	Secondary/Higher secondary	14	19
	Tertiary	7	10
Duration of using services of traditional healers	< 5 years	15	21
	5–10 years	23	32
	> 10 years	34	47

Table 4 shows the types of traditional practices for healing and black magic to harm others.

**Table 4:** Key forms and practices of traditional healers and sorcerers

Traditional healing type	Key practices
*Kalami* healing	*Kalami* healing service was offered by *Hujurs* (the religious guide in the Muslim community). *Kalami* practice required a complete code of Islamic life and also a firm belief on the Quran of the patients for effective healing.
*Bhandari* healing	*Bhandari* healers were also religious guides in rural areas but supposedly they have supernatural power; worked as human media to link up the patients with their living, central charismatic religious leader (*Baba* meaning ‘Father’) for healing purposes. The *Bhandari* practice avowed to cure any mental or physical diseases.
Spiritual healing	In spiritual healing practice, various problems of people were diagnosed and cured by making the so-called communication with dead pious ancestors or diverse sacred spirits or gods through meditation, and offering torturous treatment. Although the consultation and diagnostic fees were very little, the spiritual healing required different types of treatment instruments, which made the treatment implicitly expensive; sometimes, more expensive than the modern biomedical diagnosis and treatment.
Sorcery	The sorcerers used the so-called supernatural power to harm others in unethical ways; they also claimed they could cure all sorts of spiritual/mental disorders, offering black magic and even abolish the effectiveness of sorcery by others, upon secret requests with higher fees
*Kabiraji* healing	*Kabiraji* was the most widely-practiced traditional healing system in rural areas and was based on available medicinal plants, and substances of animal origin, which often treated patients like formal doctors, using both indigenous knowledge and modern laboratory facilities as well, and helped in curing physical diseases while symptoms of ailment were noticed. The government-approved Unani and Ayurvedic practitioners were also used to be called *Kabiraj* in some localities.
Home Medicine	Home Medicine inspired rural people to stay at home for minor disorders; instead it suggested that they use known medicinal plants and other home essentials as medicine based on their inherited experience.

### Types of traditional healing practices and their major differences

The study identified mainly two types of traditional healing systems in the rural setting: (i) religious healing system and (ii) non-religious healing system.

### Religious healing practices

Religious healing systems included the use of verses from religious books, wishing good health for the patients; verses were usually written on a paper and given as *Tabij* (amulets). Sometimes the religious verses were recited and blown on the face or on water and food items, either to drink, to eat, or to sacrifice as offerings in the name of God, gods, etc. Religious healing practices in the study villages were termed as Kalami, Bhandari and Spiritual. Kalami and Bhandari healing conventionally used religious verses or other religious incantations in healing, and healers of these systems worked as religious guide in the society, whereas spiritual healing was mostly performed reportedly by establishing a communication with diverse sacred spirits and dead pious ancestors through meditation.

### *Kalami* healing practice

Kalami is the healing practice provided by religious leader in Muslim community based on the verses of Quran (locally called Kalam). This healing system clearly differentiates medical based diseases (physical diseases) and spiritual difficulties. Kalami healers (*Hujurs*) reported that they offer to cure problems that have no physical symptoms, like embodiment of devil entities, abolishing black magic, complexity in going abroad, infertility, and resolving family dispute. However, they sometimes also treat patients returned from medical centers.

*Kalami* healing method prioritized diagnosis of the diseases, for which some verses of the Quran were written on a paper and a *bodna* (a small kind of pitcher made of copper or aluminum) was kept on it. If the disorder was spiritual, the *bodna* would move automatically; otherwise, it would be perceived as a physical disease, and the sufferer would be advised to go to the medical doctor directly. This system of diagnosis was called ‘*bodna pora*’ (invoked *bodna*). Likewise, if a woman was incapable of giving childbirth, Kalami healing required an apple or an egg to write *‘Doas’* (supplication) on it and advised that woman to have the invoked apple/egg in the name of God. If a person was considered to be affected with evil spirits, this curing method required a bottle gourd to write some *‘Doas’* on it, and the sufferer or his/her relatives were suggested to cut that bottle gourd into two pieces after returning home. The patient was also given ‘*pani-pora*’ (invoked water) and advised to drink it. Thus, the evil power was reportedly exorcised from the affected person.

*Hujurs* commonly offered their ‘*pani pora*’ to everyone for drinking it to cure all spiritual disorders, or to fulfill one’s latent intention and demanded nothing else but money; the demanded amount depended on the financial condition of the presenting person. However, some such healers do not usually fix fees; they take whatever people offer them with satisfaction after cure. Kalami healing practices could be assimilated usually by self-practice and sheer beliefs on the Holy Quran.

A woman who benefited from *Kalami* healing method, said:

*“I do believe in the light of my experience for the last five years that Kalam (verses of the Quran) never fails, if there are sheer beliefs on its healing powers.”*

### *Bhandari* healing practice

*Bhandari* healing practice helped people to get rid of various difficulties in the name of *Dorbar Sharif* (Mausoleum) of Islamic Saint Gousul Azam *Baba* (spiritual ‘Father’) Maiz Bahandari, located in Chittagong district of Bangladesh.

The *Bhanderi* healers claimed to have a miraculous power in flying invisibly and could go to the Heaven, if they just wanted this. They usually have a peculiar outer appearance, wreathed in many necklaces (sometimes made of odd materials), each of which was said to be spiritually significant to them. They first diagnosed the disorder in meditation with the assistance of ancestral *Baba* in their healing room, where a chair was reserved for *Baba,* with other peculiar instruments.

In *Bhandari* healing system, a paper with reportedly Persian scriptures was used commonly; this was too obscure for lay people to understand. The paper was put inside water contained in a pot. It was firmly believed that, if anyone could drink that water faithfully, with the name of Gousul Azam *Bab*a Maiz Bahandari, all the spiritual disorders would be cured, along with the physical sickness, whether it was cancer, AIDS, or any other complex ailment.

This paper with Persian scriptures was also provided as *Tabij* (amulets) to use for different diseases. This invaluable spiritual paper was said to be brought from the Rouza Sharif (Mausoleum) of Khawza Najim Uddin Chisti (a famous and well-respected Islamic religious leader in the Indian Sub-continent). The *Bhandari* healers demanded nothing for service; they took whatever people gave with satisfaction. Knowledge on *Bhandari* healing was reportedly a**c**hieved by the long mentoring and blessings of another senior *Bhandari healer* or *Baba*.

A *Bhandari* healer said:

“*I was just an agricultural laborer in my earlier life, and was accustomed to going to the Dorbar Sharif in Nazirhat, Chittagong, where I devoted myself sporadically in serving Baba Ziaul Haque Maiz Bhandari. Once upon a time, Baba benevolently called me nearer to him and suggested that I open my mouth, and he just infused me with blessings. Afterwards, I was spiritualized day by day, and got the healing power.”*

*Bhandari* healers were found to be full-time practitioners and demonstrated their lifestyles in different ways. They practiced meditation; some of them sang Maiz *Bhandari* songs that sometimes contradicted with the prevailing religious beliefs of the surrounding local people. Respondents reported that people, who were usually followers of *Bhandari* rituals and belief systems and also had faith enough on the *Bhandari* healing methods, were found to seek *Bhandari* healing services to address their problems.

A user of *Bhandari* treatment said:

*“I have been resolving any of the health and spiritual disorders of my all family members for the last 10 years with the Bhandari healing system; especially if my children fall sick, I just take pan-pora (invoked betel leaf) from Baba; even, sometimes, keeping Baba’s hand benignly over my children’s heads is enough to be cured.”*

### Spiritual healing practice

In spiritual healing practice, people were said to be cured by the communication of human media with diverse sacred spirits and pious ancestors; often they were offered torturous treatments, along with incantations to drive away the imaginary evil spirits or effects of sorcery or avaricious and malicious characters. Spiritual healers were locally called *Boiddya.*

The spiritual healers reported that they offered services for all spiritual problems, such as embodiment of evil entities, black magic or effect of sorcery, complexity in going abroad, infertility, resolving family disputes or village conflicts. They even often treated the chronic and complex diseases of patients, who returned from modern medical centers with treatment failure. All the women spiritual healers of the two villages were interviewed, and they belonged to the Hindu religion. The healers in this system also first tried to diagnose the disorders in meditation, uttering some words/rhymes in Hindi tunefully, and begged assistance from ‘Ma’ (particular sacred spirits calling them as ‘Mother’) and other pious ancestral spirits to diagnose the problem and cure a person.

The healers tunefully asked the patient’s name, context of the problems, and then diagnosed the types of difficulties and the necessary background at time of meditation, for which the healers required only BDT 5–20. These healers offered their healing services Saturday and Tuesday only. Although the diagnosis and consultation fee was small, the spiritual healing required a list of treatment instruments, which made the treatment implicitly expensive, sometimes even more expensive than the modern diagnosis and treatment procedures.

Majority of the patients in spiritual healing were also women. Although the healers in this system worked usually with the assistance of spirits and gods, they showed their charisma to the patient, perceiving his/her personality. If most of the descriptions of the disorder were similar with the patient’s reality, he/she delightedly said, ‘yes, yes’, ‘*Boiddy*a was right’. Spiritual healers believed that not everyone may get such a supernatural power without having some human attribution, and a person would never be eligible to assimilate the spiritual power of healing if the gods or other pious ancestral sprits did not like him/her. A Hindu woman healer in Bhabanipur village recalled:

*“I got this supernatural power in healing thirty seven years ago. At that time, I just acted as mad for one or two months, my ancestral spirits chose and compensated me, and ultimately from then on I possessed the spiritual power of healing.”*

By participatory observations, it was recorded that a large number of peculiar things were prescribed to make one cure. For example, water from seven ponds, seven eggs, 1.75 kg rice, nine candles as well as BDT 390 were prescribed for curing the headache and resolving family disputes of a single patient. This healing method also offered *‘dab-pora’* (invoked green coconut), ‘*pan-pora’* and *Tabij*’ for curing the disorder. Even though the spiritual healing system was a bit expensive to pursue in the rural context, a good number of people were found to come to the spiritual healers on healing days (Saturdays and Tuesdays). Candidates for spiritual healing considered this practice to be fruitful based on unquestionable faith.

A spiritual healing-seeker opined:

*“We don’t go to the doctor for a little case of disorder. If we are ailing, we first go to the spiritual healers. It was 6 or 7 years back, once my father was disordered suddenly, then repeatedly vomited and gradually got moribund. Afterwards, he was admitted at Niramoi Clinic in Chittagong town, kept in the clinic for about 20 days, diagnosed the whole body according to the doctor’s suggestions but nothing was effective for him, and he was just going to die. Then, we just took my father to a Hindu woman spiritual healer in the Bania Para of Bhabanipur. She could first diagnose that my father was attacked by sorcery (locally called ‘baan mara). Afterwards, she gave him pani-pora, dab-pora, and Tabij to use. After that, within one week, my father got complete recovery. From then, we firmly believe in spiritual healing.”*

The study observed that people in the community sought cures for any health and spiritual difficulties, without considering the religious perspective of the healers; often the Hindus went to the *Hujurs,* and the Muslims approached the Hindu healers to seek traditional healing.

A regular Muslim traditional healing-seeker opined:

*“We never differentiate the healers by their religion, though majority of healers in the village are Hindus. They are very cordial to address any of our problems, whenever we feel them, and they come to our home even at night in emergency, and we also attend at many of their social functions.”*

### Non-religious healing practices

Non-religious healing system included the use of anti-sorcery, the `reversed’ verses of the Quran or other religious books, and utilization of local medicinal plants or substances of animal origin as obvious curing tools. So-called supernatural power in some non-religious practices was used in ill-motivated and sacrilegious purposes to make explicit harm to the intended persons, domestic animals, and crops as well. Three distinctive types of non-religious practices usually available in the rural areas were: Sorcery, *Kabiraji,* and Home Medicine. Sorcery deliberately used the so-called supernatural power to harm others upon request with higher fees, offering black magic or anti-sorcery for healing. *Kabiraji* practice was based on local medicinal plants or substances of animal origin. *Kabiraji* practice had both formal and informal aspects: the registered traditional practitioners of Unani and Ayurvedic medicine were also locally termed *Kabiraj*. However, the informal use of wild plants for therapeutic purposes by the *Kabiraj* commonly prevailed in rural areas. Home Medicine practice involved self-medication using indigenous knowledge and items available in the home environment.

It is noteworthy that both religious and non-religious healing practice in some cases seemed to be based on a system of belief in the supernatural; the boundary between these two healing practices is in use of religion. Usually, religious healing methods use supernatural power positively for the well-being of the people, while non-religious healing systems do not use religion for healing except sorcery. However, sorcery uses so-called supernatural power for evil-intended and irreligious purpose to make explicit harm to others. It uses reversed verses of sacred religious books, which is considered completely anti-religious work (great sin), and thus forbidden in all religions in both villages.

### Sorcery

A sorcerer is someone who uses supernatural power to harm others upon secret request by someone; a sorcerer offers either black magic or anti-sorcery procedure for healing. Assimilating sorcery was said to have been difficult; sorcerers in the Muslim community in the village thought that the person who intended to learn this method had to maintain 17 conditions, which were the fundamental regulations of Islam, to achieve the power of this traditional healing system. Practice of sorcery is often locally termed as *Baan-mara.* The sorcerers reportedly cured all sorts of spiritual disorders, and abolished the effectiveness of sorcery by another; they also knew well how to enchant and attack by the magic, and performed their work, uttering some words/rhymes, incantation and antagonistic verses of a religious book. Diagnosis of the disorder was prioritized in this healing method, in which sorcerer uttered an incantation on a string, and if it was enlarged automatically, the disease was considered as spiritual, likely treatable by the sorcery. The demand for sorcerers was very high in cases of conflict over the issues of land, domestic animals, village politics, or disputes among the villagers. If a person was attacked by *Ban-mara (*sorcery), anti-sorcery was said to be the only way of curing.

A healthcare-seeker victimized by a suspected sorcery said:

*“I am a professional rickshaw-puller and the inhabitant of Boruapara in Jobra village; I consider traditional healing as the system for uneducated and poor people, as modern medical treatment is quite expensive. Few years back, I was the victim of black magic because of a conflict over land property with neighbors. When I was about to die, my family took me to a Boiddya (spiritual healer) in Rangamati. Then the Boiddya uttered some spiritual words/rhymes on a needle and pushed it into my muscle but never brought it out. Immediately, I was cured. That’s why, if I feel any spiritual difficulties, I straightly go to the Boiddya in Rangamati.”*

The sorcerers reported that they generally provide ‘*Tel pora’* (invoked oil) to massage and *Tabij* to put on the neck hanging. They can perform the contagious magic, with no direct contact needed with the victim. They required something that had been in contact with that person, such as hair, nail-parings, urine, or a bit of clothing; then mystic words or songs are recited over it. More importantly, sorcery was said to be carried out against animals or crops in the rural areas. The sorcerers accomplished their work with usual bargaining, and they demanded money to acquire valuable local instruments, like *‘Mesko, Timber, Kustori, Golapjal, and Jafran’,* which are used as ink for writing scriptures for the *Tabij.*

### *Kabiraji* healing practice

Healing based on medicinal plants, creepers, and herbs as well as locally-available substances is called *Kabiraji* healing, and both formal and informal practitioners are locally called *Kabiraj*. *Kabiraj* were considered to be successful in curing some physical diseases, using indigenous knowledge based on locally-available wild medicinal plants. There were rich floras of medicinal plants in the village. Out of the estimated 5000 species of different plants growing in the village-side, more than a thousand were regarded as having medicinal properties. The study observed that these plants were used for therapeutic purposes in both informal and traditional practices at Bhabanipur and Jobra villages. Participants reported that continuous use over long periods of time of the local plants in *Kabiraji* healing practice had made these an integral part of the culture of the people in the rural community. As a result, even at this age of highly-advanced allopathic medicine, a large number of the rural population still prefer using *Kabiraji* healing method to treat most of their diseases.

A longtime follower of *Kabiraji* healing practice opined:

*“I always approached the Kabiraj of Jelepara (East part of Bhabanipur village) whenever I feel difficulties with my children, and any of my family affairs, as I simply consider that, when medical treatment was not invented, people got cured using medicinal plants. I believe without reservation that all the treatments are within the plant creepers and herbs, which we often do not understand.”*

Some medicinal plant components that are used in the *Kabiraji* practice for curing various diseases were reported to be the sap of *Tulshi* leaves for coughing, the sap of *Neem* leaves for malaria, the juice of raw watermelon for typhoid, and the sap of mango seed with ginger for diarrhea. The *Kabiraj* in village sometimes had knowledge of modern herbal and allopathic medicines, too. The *Kabiraji* was a system of traditional healing, accepted formally by the appropriate authority of the Bangladesh Government with institutionalized form as Unani and Ayurvedic medicines that latter use modern laboratory facilities in the treatment and are usually found in the peri-urban and urban areas.

### Home medicine

The study reports that some of the villagers from every religion in the rural community were found to be conservative and preferred not to go to the traditional healers or doctors for minor illnesses. Sometimes they practiced self-medication and tried to be cured by using known medicinal plants, creepers, or something else, which were available in home environment; they termed these as home medicine. This system of healing was based on indigenous knowledge that was passed from one generation to another.

One of the home medicine-user opined:

*“We have received from our forefathers a lot of indigenous knowledge on healing using local and available instruments surrounding the home and also came to know that we should not go to the doctors for minor ailments; rather, we should prevent disease by physical activity.”*

Practitioners of home medicine could diagnose the problems using their insights and experiences and could solve minor health problems using self-treatment, which was a cost-effective practice at the family level.

### Who goes to the traditional healers and when

This study reports that people from all religions first went to the traditional healers; if not cured, they moved to medical centers for treatment using scientific methods of curing. Nevertheless, some ailments due to invisible entities were considered impossible to be cured by medical technology, and all such cases eventually had to go to the traditional healers. Individuals who were affluent, educated and aware rarely went to the traditional healers first. People with such backgrounds even felt ashamed and were insulted when asked to provide any sort of information about their going to the traditional healers, despite living as a close neighbor of the healers. On the contrary, those who were illiterate, unemployed, less aware and, more importantly, poor, usually went to the traditional healers because they could not afford modern medical treatment. However, it was observed that people of all classes and backgrounds sought assistance of traditional healers to resolve health problems that could not be treated successfully by the modern medical system.

An affluent Buddhist man in his village opined:

*“I think traditional healers themselves go to the medical center, if they can afford, for medical treatment, as traditional healing often does not use scientific methods and cannot guarantee curing the diseases.”*

An affluent Hindu woman mentioned:

*“How can you rely on them when you see they (traditional healers) are not using appropriate diagnostic measurements to identify the disease?”*

A middle-aged woman from a poor Muslim family claimed:

*“I have been benefited asking the help from Boiddya* (traditional healers) *in any kind of health disorders or personal and family difficulties for the last 20 years, and I have unquestionable faith in Bhandari healing and its Pani pora* (sacred water).*”*

A destitute man from Buddhist origin added:

*“I felt quite healthy whenever I had been to the Boiddya* (the traditional healers).*”*

### Services for disorders offered by the traditional healers

Most of the traditional healers at the village level claimed of having miraculous power in healing; they tried to persuade people to use their services. However, the study found, in participatory observations, that they used their charisma, perceiving patients’ personalities, and paying only cursory attention to the verbal history from those persons. All the healers seemed to differentiate between medically-curable diseases and those to be cured by traditional healing systems. Generally, they did not deal with ailments that had clear-cut physical symptoms and were deemed easily curable by going to the medical doctors. The study observed that healers, especially those practicing religious methods, could ensure curing those disorders that were said to be beyond natural phenomena, and were invisible, mental, spiritual, and had treatment failure at the medical centers. Above all, the study identified the complexities that the traditional healers could deal with; these involved embodiment of evil spirits, black magic or sorcery, litigating conflicts, barriers in going abroad, compromising family disputes, incapability in childbearing, pre-obstruction of intruding evil spirits as well as fulfillment of one’s latent intentions. Kabiraji and Bhandari practitioners in rural areas claimed to make serious attempts to cure all diseases that might include cancer, diabetes, AIDS, or any other chronic health difficulties.

### Functions of religions in healing

All patients from traditional healers in rural communities were asked about the functions of religions in healing. The most common responses revealed that they perceived religious belief to be an important aspect of healing, and most of the traditional healing practices were performed with the assistance of supernatural beliefs. However, patients’ responses regarding functions of religion in healing varied from person to person belonging to different religions.

A Hindu woman expressed her belief thus:

*“We perform a lot of traditional rituals and worships, believe in many powerful spirits or gods and believe a person may be attacked by diseases naturally or for neglecting duties to the powerful gods, and we all along maintain the rules and regulations of Bhogoban (God), if we would not like to fall into difficulties. In the extreme case of illness, we worship the gods, sacrifice various animals to the gods.”*

A Muslim man in a study village opined:

*“In spite of all precautions, human beings may be felled by illness as a natural process. Sometimes Allah Himself makes one ill to test the [faith of] persons or to forgive their sogira gonnah* **(**Minor sins as believed in Islam)*, and it is Allah who kindly helps all human beings to be cured.”*

An old-aged and experienced Buddhist woman believed:

*“We consider that a higher power controls our lives, deaths, and activities. This supernatural entity that we conceptualize as Bhogoban is capable of making human beings experience harm, disease, and other problems. I experience some disorders which can never be cured by medical science, for which one will obviously have to please Bhogoban by his/her religiously-expected behaviors”.*

### Measures to prevent illness

The study observed that when illness was considered to arise from the anger of supernatural power especially in Hindus, prevention took the form of endeavoring to remain on good terms with the gods and spirits. The belief in a relationship of minor sins with illness was also widely held among people in the Muslim communities. The study revealed that the Muslims strongly believed that adherence to the Islamic rituals and abstaining from sins, even if these are minor (Sogira) in nature, can be a strategy for to prevent diseases. Moreover, carrying either religious paper or object or keeping these in a home would protect the owners or the household members from the illness and misfortune caused by any evil spirits. This type of preventive measure was believed to work as insurance against any devil entities among the villagers. Such a protection strategy was also used for attracting good supernatural powers, which included Allah or His representative Prophets and the religious saints. Some people used *Tabij (amulets) to* attract good supernatural guardians.

An influential man in the Muslim community opined:

*“We all in the family use Tabij* (amulets) *that are based on sacred Kalam (verses of the Quran), and these safeguard our good fortune by protecting us from any unknown hazards.”*

### Similar attitude and characteristics of all traditional healers

Despite some differences, similarities were observed in the attitudes of traditional healers. These similarities were:

- Most of the healers differentiated between physical diseases and spiritual diseases. However, they initially tried to cure all diseases and did not suggest going to medical centers until they completely failed.
- In the course of healing, some healers were used to causing harm to others for money, but they rarely admitted it, particularly whenever they were questioned by an outsider.
- All healers tried to make their activities rational and soothing, which they thought was necessary for human life. When one approached them, they displayed their charisma. They thought their traditional healing should be more valued and that they should have an association, as the government often pressured them for using unauthorized practices. However, when observed intensively, they rarely wanted their children to follow their profession as healers. They were not satisfied personally with such a profession.

## Discussion

Traditional medicine is often the first line of treatment-seeking, particularly among low income segments of the population in rural Bangladesh. The present study mainly focused the various types of traditional healing practices, especially Kalami healing, Bhandari healing and Home medicine, which are dominant in rural areas of Bangladesh. These have rarely been found in earlier studies. Further, it appears that this is the first study of its kind to explore the diagnostic and prevention methods and functions of religion in traditional healing and to show who and when goes to traditional healers in the rural context. Previous studies conducted in Bangladesh have usually addressed only a single aspect of traditional healing or a case study. In contrast, this study investigated the basic features of all possible traditional healing practiced in rural Bangladesh.

Almost all people in rural areas included in this study followed the traditional system for healing and continued to use traditional medicine at any stage of illness due to their faith in traditional medicine and the scarcity and expense of modern medical treatment. The popularity of these healing practices shows no decline; we believe it has continued almost unchanged for generations. Similar results reported in another study of Bangladesh suggested that, at this age of highly-advanced allopathic medicine, a large majority (75–80%) of the population in this country, particularly in the rural and semi-urban areas, still prefer using traditional practice to treat of most ailments, even though modern medical facilities may be available in the neighborhood [4]. Our results were also in agreement with another study conducted in South Africa that reported that, due to high levels of poverty, traditional medicines are considered essential for the physical and mental welfare, especially among the black population in rural households in South Africa, with more than 60% of all healings taking place outside the formal medical system of western-style care [19].

Other studies in developing countries, particularly in Bangladesh [20] have also documented that trained doctors are not readily available in the rural areas; most of the childbirths are being conducted by traditional healers. They are not as skilled as trained medical practitioners are, but, in areas where there are no trained doctors, traditional healers are the only ones providing services. Among the traditional medicine user countries, the majority followed the Chinese, Indian and European traditions [21]. In the industrialized countries, almost half of the population now regularly uses some form of traditional, complementary and alternative medicines (TCAM), (United States 42%; Australia 48%; France 49%; Canada 70%), and considerable use exists in many developing countries (China 40%; India 70%; Chile 71%; Colombia 40%; up to 80% in African countries) [22–25]. These figures are not surprising, as traditional medicine continues to gain popularity both in the developing and developed countries for its proven safe and effective techniques in healing, which can contribute significantly to public health [8].

In some cases, people treated traditional healers as disreputable because they were considered uneducated and following archaic methods; sometimes they were condemned for exercising using deception in their healing strategies. Among traditional healing practices, *Kabiraji* was the most widely-practiced in rural areas based on the available medicinal plants and substances of animal origin. *Kabiraji* practice also included Unani and Ayurvedic medicine, varying from other non-botanic and religion-based traditional healing, and complementing the laboratory-based modern pharmaceuticals. Similarly, a study based on the rural Kabiraji system [26] showed that a number of plant components used by the *Kabiraj* are considered Ayurvedic medicinal plants and their usage by the *Kabiraj* matches the known Ayurvedic uses. This observation suggests that some interactions have taken place over the centuries between Ayurvedic physicians and folk medicine practitioners, which is not surprising, considering the fact that the two systems have existed side-by-side for millennia.

This study explored the *Bhandari* and *Kalami* healing practices as distinct systems in rural Bangladesh. Both *Bhandari* and *Kalami* healers worked as religious guides in the society; both usually used verses of religious books for healing disorders that were physically non-symptomatic and could not be handles by medical sciences. *Kalami* healing practice required that healers maintain a complete code of Islamic life and that patients have a firm belief in the Quran for effective healing, especially in spiritual problems, like exorcising evil spirits, making children submissive to the parents, or fulfilling one’s latent intention. *Bhandari* healers were confident they could heal any kind of disorder, including cancer and AIDS. *Bhandari* healers were said to have supernatural power, worked as human mediums to link their living and ancestral charismatic religious leaders (Baba) for healing purposes. These two types of healing systems made people think positively about curing disorders and indicated that unquestionable faith in healing systems can help people to be cured from any health problems. Similar use of the verses from religious books for medical purpose was also observed in a case study on a healer [27]; the study was conducted in a village of Narsingdi district of Bangladesh; it found a group of practitioners (including the Muslim Imams in mosques and Hindu priests in temples) who practiced other forms of medical treatment, including regular use of amulets, numerological charts and graphic designs, worship, and incantations, and which consisted of wearing, reciting, and even drinking religious texts written in water-soluble materials. However, that study only explored the use of religious texts in Kalami healing practice based on a single case study. Therefore, it could not show varied forms of healing methods. Although *Bhandari* healing practice is also dependent on religious text to some extent, the current study found that it uses many distinctive diagnostic and curing methods, which have not been reported in the earlier studies conducted in Bangladesh.

The current study reported that sorcery used supernatural power intentionally and non-religiously to harm others, claimed to cure all sorts of spiritual disorders by offering black magic. Sorcery could counteract the effectiveness of sorcery on one’s secret request with higher fees, using antagonistic verses of the religious books or uttering words/rhymes and incantation in cases of conflicts over the issues of land, domestic animals, village politics, and community disputes. These findings suggest that sorcery was conventionally disliked in all religions in rural communities, but that sorcery and anti-sorcery often strongly influenced the life of rural people. In this regard, the current study tended to agree with another study in rural Bangladesh, where it was recorded that sickness caused by a sorcerer is considered most dangerous by rural people. A sorcerer or an evil person makes incantations on tangible objects and his spells cause an immediate effect on the targeted victim [28]. Nevertheless, that study could not identify the social aspects of sorcery and its diagnostic procedures. That is, if witchcraft or sorcery was believed to be the major causes of illness, the strategy of prevention might also consist of the attempt not to arouse anyone’s anger or enmity. The fear of witchcraft or sorcery was, therefore, a positive factor in that it tended to foster harmony in interpersonal relationship among the people in rural communities.

Very limited research has been conducted on the contemporary spiritual healing practices in the context of rural Bangladesh. This study observed that various problems of the people in spiritual healing were diagnosed and cured by the communication with dead pious ancestors or diverse sacred spirits or gods, offering torturous treatment. Although the diagnostic fees in spiritual healing practices were very little, some of the instruments required for the healing made the healing system expensive. In contrast, it was observed in many parts of the world that nothing is expected from the patients, except perhaps openness to what may happen, and a degree of trust in the spiritual healing medium [29].

The extensive use of inherited indigenous knowledge still worked in the rural areas as home medicine in coping with many health problems in the home environment, making it an integral part of the local culture. This implied that people in the rural areas simply get rid of many health problems, using their insights and experiences with the help of known medicinal plants, herbs, and other home essentials. However, as far as learned, there was no other study in Bangladesh that focused on the home medicine or home remedies based on the locally-available substances in the rural context. Similar finding was, however, recorded in another study in the state of Kerala, India, where it was shown that the more extensive knowledge of some families was enough to fulfill almost all of a family’s primary healthcare needs, and was able to treat everything effectively— from common injuries to kidney stones. Knowledgeable family members were excited to share their knowledge, hardly needing to be prompted with any questions [30].

While the importance of traditional medicine has been studied in the USA, New Zealand, India, and many other countries, there has been little attention paid by researchers to the role of traditional healing practices in Bangladesh [7]. The national health policy of Bangladesh has not considered the use of traditional medicines, except Unani and Ayurvedic medicine [31]. Many countries have already developed standard national classifications and terminologies for the use of traditional medicine [7]. Bangladesh is far behind in developing a complete classification and principles to control the practices of traditional medicines. In addition, there are no directories indicating how many types of traditional means and items are used for treating any specific diseases or spiritual problems in rural areas of Bangladesh [7]. The scope of usage suggested by WHO’s recently completed survey among its Member Nations that discovered that 82% of the world’s population uses some form of traditional medicine (TM) [32].

This study conducted a qualitative investigation into some traditional healing practices in Bangladesh rarely covered in other studies; it has covered possibly all forms of traditional healing practices, along with sorcery for doing harm to others, noting their effectiveness and drawbacks. The findings offer a scenario of how traditional healing systems work as primary healthcare in rural areas and how religion, cultural beliefs, indigenous knowledge and faiths are involved in the system of traditional healing that has continued for generations. The findings of the study would suggest end-line information to the policy makers, donor agencies, NGOs, and international organizations to handle traditional medicine effectively and establish an integrated and improved primary healthcare system for rural Bangladesh.

### Limitation and strengths of the study

The clearest limitation of this study was the low number of unstructured interviews conducted in the two adjacent villages. However, this was compensated by the information obtained from participatory observations and KII. It cannot be claimed that saturation was reached or that the information obtained represents all of Bangladesh in terms of traditional healing practices. However, findings obtained from this investigation provide a concise description of prevailing traditional healing practices in the rural Bangladesh context.

## Conclusions

Both religious and non-religious healing methods are widely-practiced in rural areas. The basic forms of traditional healing systems are similar to what was earlier the case. Most of these practices are serving the needs of primary healthcare among the majority of population in the study areas, where modern health facilities are not available and medical treatment is expensive. People with low socioeconomic and educational backgrounds were more likely to seek help from traditional healers in rural settings. Findings of this study can guide development of a systematic study of traditional healing practices, in broader contexts, to sort out the national classifications for the means of using traditional medicine and produce a cost-effective and integrated healthcare system for rural population in Bangladesh. It can also help policy-makers and health experts think about the effectiveness and significant role of traditional medicine and to formulate the principles and regulations in traditional healing systems as a WHO-prioritized area. However, in the absence of supervision from the appropriate authority, traditional healing practices may sometimes worsen the sickness or cause harm to the users.

### Recommendations

Based on the findings of the study, the following recommendations are made for the traditional healing systems and the practitioners:

1. An autonomous body of traditional medicine (TM) is needed in the Ministry of Health and Family Welfare under which tradition healing practices and types should be registered, and the principles of traditional healing practices and their code of conduct be clearly defined. This will save costs of health services, as many people use services of traditional healers for their primary healthcare.
2. All government-approved traditional healers should receive basic training so that they can understand the danger signs of major critical diseases and be able to refer to expert doctors when required.
3. Traditional healing practices should be subjected to inspection and be penalized, and potentially punished, in cases of sorcery, or false or malpractices in traditional healing.

## Abbreviations

KII: Key Informant Interview
NGO: Non-governmental Organization
PHC: Primary Healthcare
T&CM: Traditional and complementary medicine
TCAM: Traditional Complementary and Alternative Medicine
TM: Traditional medicine
WHO: World Health Organization
